# All Inside Intraepiphyseal ACL Reconstruction Using Flexible Curved Instrumentation and Intraoperative Fluoroscopy in a Skeletally Immature Patient

**DOI:** 10.1155/2021/3956524

**Published:** 2021-04-21

**Authors:** Richard N. Puzzitiello, Avinesh Agarwalla, Grant Garcia, Enrico M. Forlenza, Brian Forsythe

**Affiliations:** Midwest Orthopaedics at Rush, Rush University Medical Center, Chicago, IL, USA

## Abstract

**Case:**

A 13-year-old skeletally immature female presenting with an anterior cruciate ligament (ACL) rupture after a noncontact injury was treated with an intraepiphyseal ACL reconstruction. Flexible instrumentation was utilized to drill a femoral tunnel with an anatomic starting point, with a trajectory that curved inferolaterally away from the physis. At three years postoperatively, she had returned to her preinjury functioning and did not display any lower limb length growth abnormalities.

**Conclusions:**

The novel application of curved guides and flexible instruments, with intraoperative fluoroscopy, facilitated growth plate avoidance and a successful outcome of ACL reconstruction in a skeletally immature patient.

## 1. Introduction

Anterior cruciate ligament (ACL) injuries in children and adolescents have become increasingly common, in part due to increases in youth sports participation [1, 2]. ACL reconstruction in a skeletally immature patient demands special considerations, as violation of open physes during tunnel drilling may produce growth disturbances [3–5]. Although some studies have shown excellent outcomes and high levels of return to sport after transphyseal ACL reconstruction [4, 6], many authors suggest physeal-sparing methods to avoid causing growth disturbance [2, 3, 5–8].

The authors of this article hypothesized that it would be possible to create an anatomically placed femoral tunnel that does not violate the physis using a curved guide and intraoperative fluoroscopy. This proposed method would avoid the risk of growth abnormalities with transepiphyseal techniques, while minimizing the risk of nonanatomical tunnel placement previously described in intraepiphyseal reconstruction. The purpose of this report is to describe the case of a pediatric ACL reconstruction using curved instrumentation and intraoperative fluoroscopy to create an anatomically placed femoral tunnel that does not violate the physis. Informed consent and assent were obtained from the patient and her legal guardian for reporting of this case.

## 2. Case Presentation

A 13-year-old female reported to clinic for evaluation after sustaining a right knee injury five months prior. The patient was 5'2”, 45.3 kg, Tanner Stage 4, and she was 13 years old at age of first menses, and her parents were 5'4” and 5'10”. She reported a hyperextension noncontact injury playing soccer. On exam, she demonstrated full range of motion bilaterally, 2B right Lachman exam, and a right 2+ pivot-shift.

**Figure 1:**
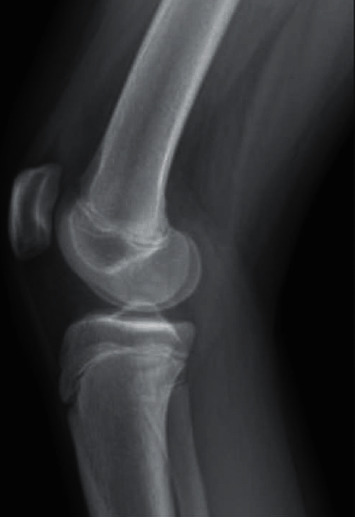
Preoperative lateral view radiograph of the right knee demonstrating open growth plates.

Radiographic imaging was obtained of the right knee demonstrating open physes (Figure 1), and an MRI demonstrated complete tear of her ACL with associated bone contusions. After discussion with this patient and her guardian, it was collectively decided to proceed with arthroscopic ACL reconstruction with a hamstring autograft. The plan was to proceed with an intraepiphyseal ACL femoral fixation technique to minimize the risk for potential growth abnormalities.

## 3. Treatment and Surgical Technique

**Figure 2:**
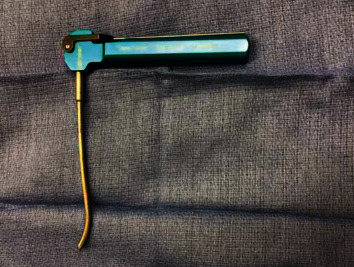
Curved endoscopic femoral drill guide (Stryker, Kalamazoo, MI).

**Figure 3:**
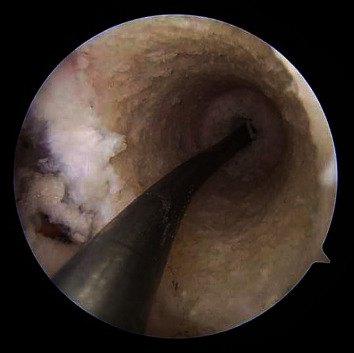
Arthroscopic view of the flexible guide pin in the femoral tunnel, with no visible physeal cartilage observed.

**Figure 4:**
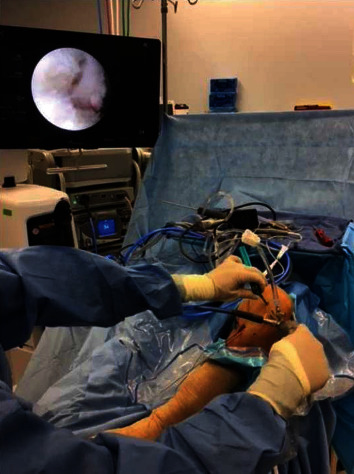
Intraoperative photo demonstrating the positioning of a curved endoscopic femoral drill guide, aimed at approximately 40° angle above the horizontal plane.

**Figure 5:**
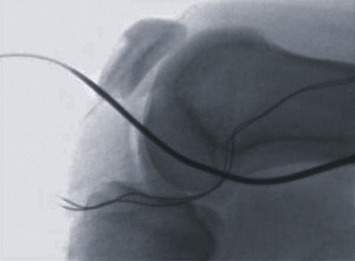
Intraoperative lateral fluoroscopic image confirming the placement of the flexible guide pin below the physis.

**Figure 6:**
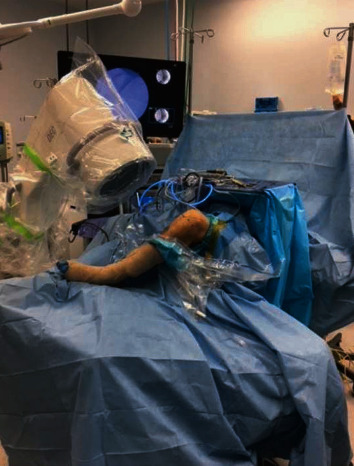
Intra-operative image of a C-arm positioned to obtain fluoroscopic imaging of guide pin for the femoral tunnel to ensure intra-epiphyseal placement.

**Figure 7:**
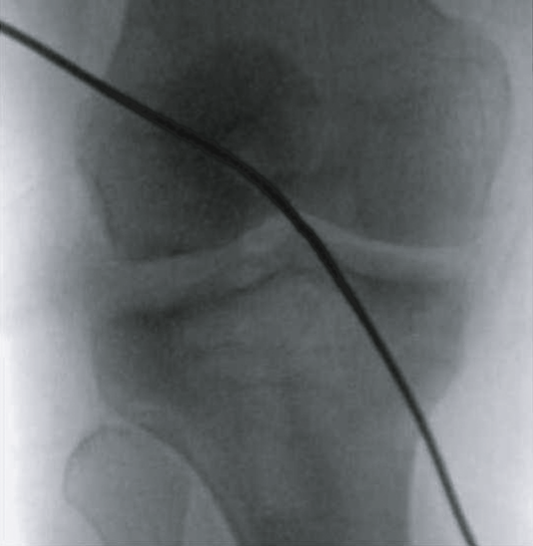
Intra-operative anterior-posterior fluoroscopic image confirming placement of the flexible guide pin below the physis.

The procedure began with autologous hamstring tendon harvests, gracilis, and semitendinosus, which were prepared in a standard quadruple bundle fashion. Each bundle was passed through a 10 mm EndoLoop button (Smith & Nephew, London, England) for fixation purposes. Diagnostic arthroscopy was notable for intact cartilage surfaces of the medial and lateral patellar facets and trochlea. The intercondylar notch was then inspected, and a positive empty wall sign was noted with complete avulsion and marked attenuation of the ACL tissue. The site of the most isometric location for femoral tunnel placement was marked at the intersection of the bifurcate and intercondylar ridges on the inferior one-third of the MWLFC wall. A curved endoscopic guide and Versitomic flexible reaming system (Stryker, Kalamazoo, MI) were used for this technique (Figure 2). The curved guide was advanced through the AM portal and positioned on the marked location. The primary advantage of these instruments is that they allow for recreation of the ACL footprint while optimizing tunnel length and avoiding the need for hyperflexion of the knee [9, 10]. The previous techniques describe placing the curved endoscopic femoral guide in the middle of the femoral footprint at 45° below the horizontal access, resulting in a superolateral trajectory. This trajectory crosses the femoral growth plate and results in an exit point along the anterolateral thigh [9]. In the current technique, the guide was positioned to achieve an inferolateral trajectory (Figures 3 and 4). This was achieved by rotating the guide an additional 85° clockwise from the previously described techniques' trajectory, so that the guide is 40° above the horizontal plane (Figure 4). With the guide in this position, the knee was placed in 110° of flexion, and a 2.2 mm flexible guide pin was advanced until it exited the femur (Figure 5). The trajectory created by this additional rotation of the guide prevents the guidewire from crossing the physis. Fluoroscopic imaging was used throughout the guidewire advancement to ensure that the physis was not violated. A 4.5 mm drill bit was then utilized to perforate the cortex, creating a tunnel length totaling 35 mm. The tunnel was drilled slowly to prevent possible heat damage to the growth plate [11] [12]. A C-arm was used incrementally during the femoral tunnel drilling as well, to obtain AP (Figure 6) and lateral fluoroscopic views (Figures 5 and 7), confirming appropriate intraepiphyseal positioning of the guidewire without violation of the physis.

The tibial tunnel was then established in standard fashion, exiting on the tibial plateau at the center point of the AM and PL bundles along the anterior intertubercular ridge. The graft was passed across the tibial tunnel, across the notch, and into the femoral tunnel to an appropriate depth, followed by EndoButton flipping for fixation purposes. With the knee in 5° of flexion and a posterior drawer force applied, tension was applied to each of the four tendon strands. A 6 mm dilator followed by a 7 mm GraftBolt PEEK sleeve (Arthrex. Naples, FL) was then advanced to achieve fixation. A 7 mm PEEK interference screw (Arthrex. Naples, FL) was then placed within the sheath to compress the tendon tissue against the walls of the tibial tunnel and to achieve stable purchase. The wounds were then irrigated and closed in standard fashion to complete the procedure.

### 3.1. Rehabilitation Protocol

After surgery, the patient underwent a standard ACL rehabilitation protocol. Range of motion and weight-bearing was progressed immediately as tolerated. By three months postoperative, the range of motion was from 2 degrees of hyperextension to 140 degrees of flexion, which was symmetric bilaterally. Jogging was initiated at 4 months. Plyometrics and sport-specific rehab was advanced at 5 months postoperatively. At 1-year, she reported that she felt 100% of normal on her operative side. Her physical exam demonstrated a normal gait, and she displayed a symmetric single-leg hop ×5 without pain. She was then released to sport without restriction.

### 3.2. Final Follow-Up

**Figure 8:**
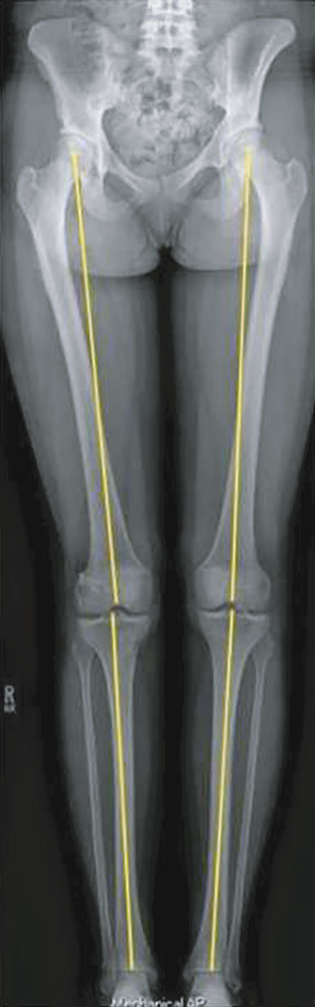
Standing mechanical axis anterior-posterior radiograph at 3 years postoperative.

At a three-year follow-up appointment, she reported a successful return to her baseline function and return to soccer and sport without limitation, further complication or lower limb length growth abnormalities (Figure 8). The side-to-side difference on KT-1000 arthrometer testing was 0.9 mm, in favor of her operative side. She had a grade 1A Lachman exam and a stable pivot-shift exam. Her IKDC, Marx, and Tegner scores at this final follow-up were 95.36, 8, and 9, respectively.

## 4. Discussion

This case report presents the treatment of an ACL rupture in a skeletally immature pediatric patient with an intraepiphyseal ACL reconstruction utilizing a flexible curved guidewire under intraoperative fluoroscopic guidance, to confirm appropriate femoral tunnel placement. Additional strategies utilized during this procedure to prevent growth plate disturbance included drilling at a slower speed to prevent heat damage to the growth plate and drilling smaller tunnels [12]. A transphyseal technique was used on the tibial side because previous studies have shown excellent outcomes with such combined methods [8, 13], and because most cases of growth abnormalities are attributed to femoral physes violation [14].

Many surgical techniques have been developed to balance the goal of restoring anatomic ACL placement while safely avoiding the physis to prevent growth risks. These physeal-sparing techniques fall into one of two general categories, extra-articular reconstruction [15–18], and intraepiphyseal reconstruction [19, 20]. Extra-articular reconstructions are favorable for their ability to prevent any growth-altering structural damage [16]; however, these techniques are neither anatomic nor isometric [21] and they may overconstrain the knee [7]. Several cases of growth disturbances after intraepiphyseal ACL reconstruction in skeletally immature patients have been described, despite the goal of this technique being to avoid this complication [13, 22]. Frosch et al. argue that this might be due to heat damage caused by drilling parallel to the physis, as well as by implant expansion resulting in a pressure effect against the physis [13]. For these reasons, the senior author of this report decided to take a novel surgical approach that would optimize anatomic placement and risk avoidance.

In adults, flexible instrumentation has been shown to create more anatomic and longer femoral tunnels that are further away from the posterior cortex compared to rigid drilling systems [23, 24]. Additionally, these results can all be obtained at lower knee flexion angles with curved instruments, making this stage of the procedure less technically demanding with less risk of complications [9, 10]. Rigid reamers have been shown to create horizontal tunnels with higher tunnel acuity, which may influence contact pressure between the graft and the tunnel aperture [25]. The authors of this report suggest that the benefits of flexible instrumentation may apply in skeletally immature patients, with the added benefit of creating a femoral tunnel that is directed further away from the growth plate. This would theoretically decrease the risk of growth plate disruption directly, along with a decrease in heat damage or pressure effect theoretically observed in tunnels parallel to the growth plate created with rigid instrumentation [13]. However, higher evidence studies are necessary to confirm these hypotheses.

The goals of this all intraepiphyseal ACL reconstruction was to restore functionality and successful return to sport, while avoiding risks of growth abnormalities. The novel application of curved guides and flexible instruments, with intraoperative fluoroscopy, facilitated growth plate avoidance and a successful outcome. This technique is a viable approach to ACL injury in the skeletally immature patient, although higher level studies are needed for further validation of its safety and efficacy.

### 4.1. Limitations

A notable limitation of this technique presentation is that preoperative KT-1000 and patient-reported outcome measures (including Marx, IKDC, and Tegner scores) were not recorded. For this reason, we were unable to draw conclusions regarding the change in these measures. However, we noted excellent outcomes for both KT-1000 and patient-reported outcome measures for this patient undergoing all inside intraepiphyseal ACL reconstruction with a flexible curved guide. Other limitations of this study include the lack of ability to generalize the findings to the broader population or establishing cause and effect relationships due to the small sample size and retrospective design. Higher-level studies are required to validate the safety and efficacy of the presented technique.

## References

[1] Brusalis C. M., Lakomkin N., Suryavanshi J. R. (2017). Clinical outcome reporting in youth ACL literature is widely variable. *Orthopaedic Journal of Sports Medicine.*.

[2] DeFrancesco C. J., Storey E. P., Shea K. G., Kocher M. S., Ganley T. J. (2018). Challenges in the management of anterior cruciate ligament ruptures in skeletally immature patients. *Journal of the American Academy of Orthopaedic Surgeons*.

[3] Chotel F., Henry J., Seil R., Chouteau J., Moyen B., Bérard J. (2010). Growth disturbances without growth arrest after ACL reconstruction in children. *Knee Surgery, Sports Traumatology, Arthroscopy*.

[4] Longo U. G., Ciuffreda M., Casciaro C. (2017). Anterior cruciate ligament reconstruction in skeletally immature patients. *The Bone & Joint Journal*.

[5] Calvo R., Figueroa D., Gili F. (2014). Transphyseal anterior cruciate ligament reconstruction in patients with open physes: 10-year follow-up study. *The American Journal of Sports Medicine*.

[6] Hui C., Roe J., Ferguson D., Waller A., Salmon L., Pinczewski L. (2012). Outcome of anatomic transphyseal anterior cruciate ligament reconstruction in Tanner stage 1 and 2 patients with open physes. *The American Journal of Sports Medicine*.

[7] Kennedy A., Coughlin D. G., Metzger M. F. (2011). Biomechanical evaluation of pediatric anterior cruciate ligament reconstruction techniques. *The American Journal of Sports Medicine*.

[8] Kaeding C. C., Flanigan D., Donaldson C. (2010). Surgical techniques and outcomes after anterior cruciate ligament reconstruction in preadolescent patients. *Arthroscopy: The Journal of Arthroscopic & Related Surgery*.

[9] Fitzgerald J., Saluan P., Richter D. L., Huff N., Schenck R. C. (2015). Anterior cruciate ligament reconstruction using a flexible reamer system: technique and pitfalls. *Orthopaedic Journal of Sports Medicine*.

[10] Forsythe B., Collins M. J., Arns T. A. (2017). Optimization of anteromedial portal femoral tunnel drilling with flexible and straight reamers in anterior cruciate ligament reconstruction: a cadaveric 3-dimensional computed tomography analysis. *Arthroscopy: The Journal of Arthroscopic & Related Surgery*.

[11] Meller R., Kendoff D., Hankemeier S. (2008). Hindlimb growth after a transphyseal reconstruction of the anterior cruciate ligament: a study in skeletally immature sheep with wide-open physes. *The American Journal of Sports Medicine*.

[12] Peterson D. C., Ayeni O. R. (2016). Pediatric anterior cruciate ligament reconstruction outcomes. *Current Reviews in Musculoskeletal Medicine*.

[13] Frosch K.-H., Stengel D., Brodhun T. (2010). Outcomes and risks of operative treatment of rupture of the anterior cruciate ligament in children and adolescents. *Arthroscopy: The Journal of Arthroscopic & Related Surgery*.

[14] Collins M. J., Arns T. A., Leroux T. (2016). Growth abnormalities following anterior cruciate ligament reconstruction in the skeletally immature patient: a systematic review. *Arthroscopy: The Journal of Arthroscopic & Related Surgery*.

[15] Brief L. P. (1991). Anterior cruciate ligament reconstruction without drill holes. *Arthroscopy*.

[16] Kocher M. S., Garg S., Micheli L. J. (2006). Physeal sparing reconstruction of the anterior cruciate ligament in skeletally immature prepubescent children and adolescents. *Journal of Bone and Joint Surgery*.

[17] Nakhostine M., Bollen S. R., Cross M. J. (1995). Reconstruction of mid-substance anterior cruciate rupture in adolescents with open physes. *Journal of Pediatric Orthopedics*.

[18] Parker A. W., Drez D., Cooper J. L. (1994). Anterior cruciate ligament injuries in patients with open physes. *The American Journal of Sports Medicine*.

[19] Anderson A. F. (2004). Transepiphyseal replacement of the anterior cruciate ligament using quadruple hamstring grafts in skeletally immature patients. *The Journal of Bone & Joint Surgery*.

[20] Guzzanti V., Falciglia F., Stanitski C. L. (2003). Physeal-sparing intraarticular anterior cruciate ligament reconstruction in preadolescents. *The American Journal of Sports Medicine*.

[21] Redler L., Meyers K., Munch J., Dennis E., Nguyen J., Stein B. S. (2016). Anisometry of medial patellofemoral ligament reconstruction in the setting of patella alta and increased tibial tubercle-trochlear groove (TT-TG) distance. *Arthroscopy*.

[22] Kocher M. S., Saxon H. S., Hovis W. D., Hawkins R. J. (2002). Management and complications of anterior cruciate ligament injuries in skeletally immature patients: survey of the Herodicus Society and The ACL Study Group. *Journal of Pediatric Orthopedics*.

[23] Steiner M. E., Smart L. R. (2012). Flexible instruments outperform rigid instruments to place anatomic anterior cruciate ligament femoral tunnels without hyperflexion. *Arthroscopy: The Journal of Arthroscopic & Related Surgery*.

[24] Silver A. G., Kaar S. G., Grisell M. K., Reagan J. M., Farrow L. D. (2010). Comparison between rigid and flexible systems for drilling the femoral tunnel through an anteromedial portal in anterior cruciate ligament reconstruction. *Arthroscopy: The Journal of Arthroscopic & Related Surgery*.

[25] Basdekis G., Abisafi C., Christel P. (2009). Effect of knee flexion angle on length and orientation of posterolateral femoral tunnel drilled through anteromedial portal during anatomic double- bundle anterior cruciate ligament reconstruction. *Arthroscopy*.

